# Impact of modern central nervous system radiation on multiple sclerosis relapse and progression

**DOI:** 10.1016/j.ctro.2026.101176

**Published:** 2026-04-22

**Authors:** Hannah H. Zhao-Fleming, Daniel K. Ebner, William Sperduto, Brady S. Laughlin, Daniel H. Lachance, William G. Breen, W. Oliver Tobin

**Affiliations:** a Department of Neurology, Mayo Clinic, Rochester, MN, USA; b Center for Multiple Sclerosis and Autoimmune Neurology, Mayo Clinic, Rochester, MN, USA; c Department of Radiation Oncology, Mayo Clinic, Rochester, MN, USA; d Department Radiation Oncology, Mayo Clinic, Phoenix, AZ, USA

**Keywords:** Multiple sclerosis (MS), Ionizing radiation, Multiple sclerosis progression, Multiple sclerosis relapse

## Abstract

**Background:**

Central nervous system (CNS) radiation is associated with primary demyelination and neurotoxicity in patients with existing demyelinating disorders. We examined the safety of modern ionizing-radiation techniques in multiple-sclerosis (MS) patients.

**Patients and methods:**

Patients with MS undergoing CNS radiation for any reason between January 2005, and August 2024 were identified by chart review. Inclusion criteria were: 1) confirmed diagnosis of MS; 2) Receiving therapeutic radiation with a dose of greater than 5 Gray (Gy) to brain or spinal cord parenchyma; 3) Clinical neurology and brain MRI follow-up available following radiation.

**Results:**

Thirty-three patients were included. Median age at radiation was 60 years (54––68 IQR) and median follow-up, 20 months (range 3–99). Radiation focused on the CNS in 15/33 (45%), 5 (15%) targeted locations adjacent to the CNS parenchyma (meningioma 4, trigeminal neuralgia 1), and 13 (39%), outside the CNS with > 5 Gy delivered to the CNS. Clinical MS course at radiation was relapsing remitting (19/33), secondary progressive (11/33), and primary progressive (3/33). Thirteen patients (39%) were on MS disease-modifying therapy. No clinical relapse or new-onset MS progression followed radiation. New T2 or gadolinium-enhancing lesions on T1 MRI occurred in 7/33. Radiation-field data were analysed with reference to location of 10 new demyelinating lesions in 7 patients. Most lesions lay outside the irradiated area (6/10). No patients with new lesions were on high-efficacy medications. Seven patients experienced increased expanded disability status scale (EDSS) pre-radiation to last follow-up by ≥ 1 point (median 1.5, range 1–7). One patient had known progressive disease while 5 patients’ increased disability was thought secondary to their radiation indication (3 glioblastoma and 2 meningioma).

**Conclusions:**

Modern-day ionizing radiation to the CNS in patients with multiple sclerosis was not associated with clinical worsening but can potentially be associated with an increased risk of asymptomatic inflammatory MS disease activity.

## Highlights

• Radiation is the standard treatment for CNS malignancies.
• Current CNS radiation therapies do not worsen clinical multiple sclerosis (MS).
• Modern radiation may increase the risk of asymptomatic MRI lesions.

## Introduction

Ionizing radiation is the cornerstone of treatment of many malignancies, including CNS malignancies such as glioblastoma, systemic malignancies such as breast or lung carcinomas, as well as non-malignant disorders including meningioma and trigeminal neuralgia [1], [2], [3]. Patients with multiple sclerosis (MS) who are treated with whole brain radiation experience higher neurotoxicity compared to those without demyelinating disorders [4]. Historical radiation often consisted of 2D and 3D fields delivering radiation antero-posteriorly or laterally with increased heterogenous radiation dose to uninvolved tissue. However, modern conformal radiation, enabled by techniques including MRI guidance, volume modulated arc therapy, and proton radiation can result in less dose to uninvolved tissue. In this study, we sought to understand the effect of modern ionizing radiation on neurotoxicity, inflammatory disease activity and progression in individuals with MS.

## Patients and methods

### Standard protocol approvals, registrations, and patient consents

The Institutional Review Board of the Mayo Clinic approved this study (IRB 16–010508). All patients, or their parents, consented to the use of their medical records for research purposes.

### Identification of MS patients who had undergone ionizing radiation

**Fig. 1:**
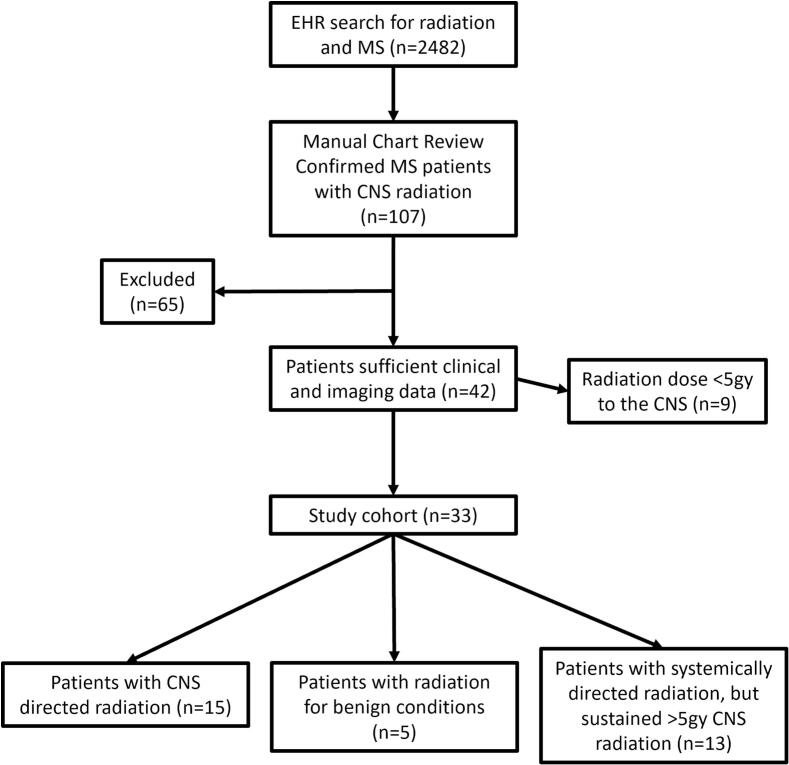
Flowchart depicting search strategy for identification of patients included in the study. Abbreviations: CNS, central nervous system; MDE, Mayo Data Explorer, an institutional electronic retrieval system that interrogates patient charts; MS, multiple sclerosis.

We used our institution's medical record diagnostic linkage system to identify all patients treated at Mayo Clinic from January 2005 through August 2024 who had a diagnosis of MS and underwent radiation (Fig. 1). Our inclusion criteria were: 1) Confirmed diagnosis of MS based on 2017 diagnostic criteria [5]; 2) Radiation delivered at a dose of 5 Gy or more to the CNS, regardless of the intended target; 3) At least one neurology evaluation and at least one brain MR image available for review, both at least 3 months after receiving ionizing radiation. For cohort identification, patients with MS were cross‑referenced against our institutional radiation oncology database and included if treatment planning dosimetry demonstrated a maximum point dose ≥ 5 Gy to the brain and/or spinal cord, structures that are routinely contoured when targets are intraparenchymal or adjacent to the CNS.

### Data collection

Demographic and clinical data was abstracted from by chart review. Disability was assessed using the expanded disability status scale (EDSS) [6]. Specific radiation treatment data were compiled using institutional treatment planning software (Eclipse ^TM^).

### Clinical and radiographic outcomes

All post-radiation MRIs, including the images and radiology reports, were reviewed independently by two neurologists (HHZ and WOT). Lesions were judged based on typical MS morphology and location, as described by the 2017 McDonald’s criteria [5]. New clinical relapse was defined as a subacute onset of typical MS symptoms, lasting for more than 24 h, with an identified associated MRI lesion in the correct location. MS progression was defined as sustained worsening of clinical symptoms over at least 12 months, in the absence of new MRI lesions. These MRIs were rigidly fused to prior radiotherapy planning scans with dose metrics delivered to these lesions identified.

For spatial lesion‑to‑field analysis, post‑radiation MRIs were rigidly registered to the original radiotherapy planning CT scans. New demyelinating lesions were contoured, and minimum, mean, and maximum dose to each lesion was extracted from the registered dose distribution. Lesions were categorized a priori based on their spatial relationship to the radiation field as follows: (1) outside field (no overlap with any irradiated CNS structure), (2) peripheral/low‑dose region (within regions receiving incidental dose < 2000 cGy outside the primary target volume), or (3) center/high‑dose region (within the primary high‑dose treatment volume). Lesion identification and contouring were performed by neurologists experienced in MS imaging review, in collaboration with radiation oncology using treatment planning software; reviewers were not blinded to radiation fields.

### Statistical methodology

Continuous variables are reported as median (range) and were tested by Wilcoxon rank sum test. Categorical variables were reported as number (%) and were tested by Fisher’s exact test. P-values < 0.05 were considered statistically significant.

### Data availability statement

Anonymized data used for this study are available upon reasonable request from the corresponding author.

## Results

### Total patient cohort

We identified 33 patients with MS who underwent ionizing radiation to the CNS (Fig. 1 and Table 1). Treatment indication was primary CNS malignancy (11/33), brain metastasis (4/33, 2 lung malignancies, 1 diffuse large B-cell lymphoma, and 1 medullary thyroid carcinoma), meningioma (4/33), trigeminal neuralgia (1/33). Lastly, 13/33 (3 breast malignancies, 2 lung malignancies, 2 squamous cell carcinomas of skin, 2 thyroid malignancies, 1 GI malignancy, 1 retroperitoneal sarcoma, 1 renal cell carcinoma, 1 parotid adenocarcinoma) patients underwent extra-CNS radiation, with some incidental exposure of brain or spinal cord > 5 Gy.

**Table 1:** Cohort characteristics.

General		Sex	26 (79%) Female
		Smoking status	Never (15, 45%), prior (15, 45%), current (3, 9%)
		Age at radiation (years)	60 (18–82)
		Demographic	30 (91%) White
Radiation Target		Primary CNS tumor	11 (33%)
		Metastasis to CNS	4 (12%)
		Extra-CNS radiation	13 (39%)
		Meningioma	4 (12%)
		Trigeminal neuralgia	1 (3%)
Baseline MS characteristics		MS symptom onset age	36 (17–56)
		MS diagnosis age	42 (17–62)
		MS progression age	14, 55.5 (42–78)
		Age when walker was required	11, 63 (54–80)
		Age when wheelchair was required	4, 58.5 (55–81)
		MS type	RRMS (19, 58%), PPMS (3/33, 9%), SPMS (11/33, 33%)
Outcome		Follow-up interval (months)	20 (3–99)
		Increase in EDSS	7 (21%)
		Median change	1.5 (1–7)
		Deceased	6 (18%)
	Cause of death	Tumor progression	3 (50%)
		MS progression	0
		Other	2 (33%)^1^
		Unknown	1 (17%)

Data regarding time is presented as (years, n, median (range)), unless otherwise specified. Abbreviations: CNS, central nervous system; EDSS, expanded disability status scale; MS, multiple sclerosis; PPMS, primary progressive MS; RRMS, relapsing remitting MS; SPMS, secondary progressive MS. ^1^Other deaths included gastrointestinal bleeding (n = 1) and coronovirus-19 infection (n = 1).

The median interval between the most recent pre‑radiation brain MRI and initiation of radiation therapy was 20 days (interquartile range 10–59 days). Two patients had evidence of MS disease activity in the year preceding radiation, including one patient with an asymptomatic enhancing brain lesion and one patient with MRI‑confirmed symptomatic transverse myelitis.

At the time of radiation 19/33 had RRMS, 3/33 had PPMS, and 11 had SPMS. In the 14 subjects with either primary or secondary MS progression, the median age of onset of progression was 55.5 years. The median age at time of radiation was 60 (range 18–82) years. Patients were followed for a median of 20 months (range 3–99). Twelve patients were on an MS disease-modifying therapy (DMT) during radiation, 4 of whom were on a high-efficacy medication (Ocrelizumab n = 3, cladribine n = 1). Other DMTs used were glatiramer acetate (2), dimethyl/diroximel fumarate (2), fingolimod (1), interferon beta (1), and teriflunomide (1). Six patients (18%) died during the follow-up period, 3 due to cancer progression. Further details of our patient cohort can be found in Table 1. In the 33 cases reviewed, radiation was administered using proton beam therapy, while 1 patient underwent X-ray therapy.

### Impact of ionizing radiation on multiple sclerosis disease course

**Fig. 2:**
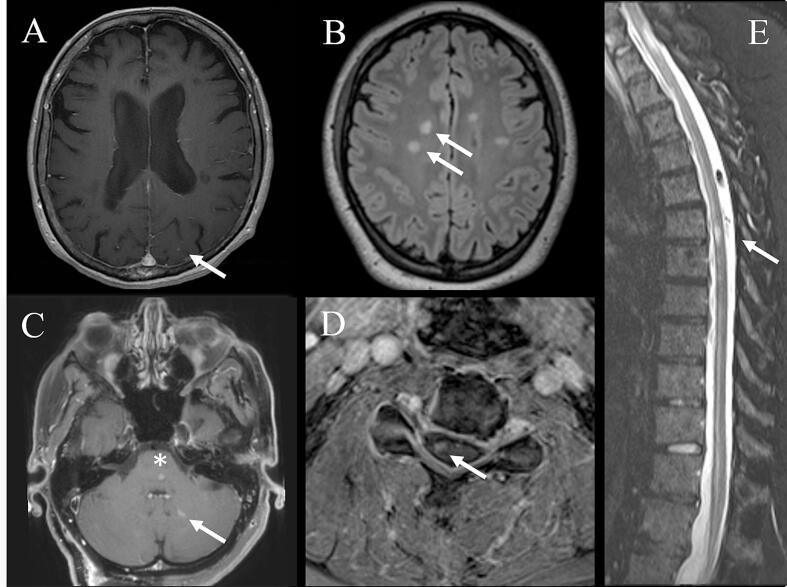
Typical new demyelinating lesions after ionizing radiation. We found both gadolinium enhancing (A) and non-enhancing (B) lesions in the bilateral cerebral hemispheres, enhancing lesion in the cerebellum (C), and enhancing (D) and non-enhancing lesions (E) of the spinal cord. Arrowheads highlight new demyelinating lesions. Asterisk on (C) highlights a persistently enhancing lesion that we deemed was not consistent with a demyelinating lesion.

No patients experienced a clear clinical MS relapse during follow-up (median follow-up 20 months [9–37, IQR]). Seven patients (21%) experienced an increase in EDSS during the follow-up period, with a median increase of 1.5 (range 1–7). One patient had known progressive disease that continued to progress post radiation. Five patients had an increase in disability thought to be secondary to their underlying malignancy (3 glioblastoma [GBM], 2 meningioma) and subsequent treatment. Two patients with GBM had progression of their glioma, leading to increasing disability before death due to malignancy. One patient with meningioma developed mild ataxia due to the meningioma and subsequent resection. Another patient suffered an acute visual loss 4 months after a *para*-clinoid meningioma radiation. Neuro-ophthalmology evaluation in the acute phase noted no disc oedema on exam and the patient had no improvement with 5 days of IV methylprednisolone. MRI shows peripheral enhancement with central sparing of the involved optic nerve. Taken together, this was thought to be more consistent with radiation induced optic neuropathy as opposed to optic neuritis. Seven patients (21%) developed new asymptomatic brain MRI lesions following radiation, with 1 patient developing new enhancing lesions in three separate imaging studies over four years. New demyelinating lesions were most observed in the cerebral hemispheres (5/7) but also occurred in the cerebellum (1/7) and the spinal cord (2/7). More than 1 new lesion was noted in some patients. In 5/7 patients, new lesions demonstrated gadolinium enhancement, while 2/7 patients had new non-enhancing T2 hyperintense lesions. Representative images of these new lesions are depicted in Fig. 2.

**Fig. 3:**
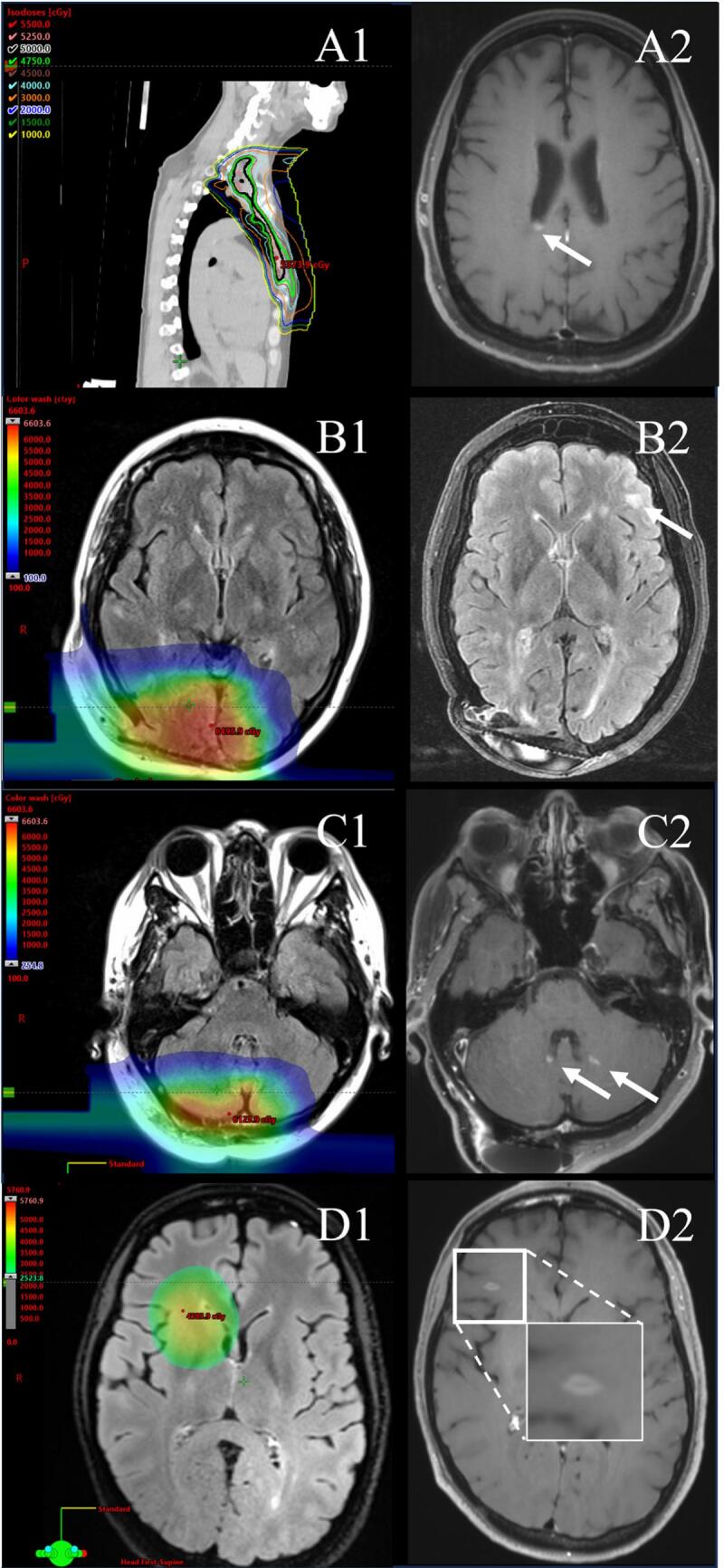
New demyelinating lesions have varying overlap with the radiation field. We found new demyelinating lesions either distant from (A), near (B), at the periphery of (C), or within (D) the radiation field. A1-D1 depicts the radiation fields while A2-D2 depicts the new demyelinating lesions. Arrowheads highlight new demyelinating lesions. Blue inset in D2 show a magnified view of a positive central vein sign.

Radiation fields from 10 progressive lesion regions, encompassing 7 patients were available for analysis. New demyelinating lesions were found outside the radiation field in 6/7 patients. Of these, 2/6 patients were radiated in the spinal cord and demonstrated new lesions in the brain (Fig. 3A), 1/6 patients was radiated in the brain and demonstrated new lesion in the spinal cord, 1 patient was radiated in the occipital lobes and had a new lesion at C4-5, 1 patient was radiated in the occipital/parietal lobes and had a new lesion in the right frontal lobe (Fig. 3B), and 1 patient was radiated in predominantly right occipital/parietal/temporal lobes and had 2 new lesions of the left frontal lobe. New demyelinating lesions could also be found in the periphery/low dose region (n = 3, Fig. 3C) or the centre/high-dose region (n = 1, Fig. 3D). Further details of the descriptions of the lesions and the clinical circumstances around them can be found in Table 2. Age of radiation has been generalized to a range of 5 years to anonymize the data.

**Table 2:** Patient level description of new demyelinating lesions after ionizing radiation.

Patient	MRI scans	*Age at radiation (years)	Lesion description	Target of radiation	Max dose radiation (cGy)	Mean dose radiation (cGy)	Location of lesion in relation to radiation field	Time between radiation and new MS lesion (months)	Medication during radiation	Medication during lesion
1		55–60	T2 hyperintensity of cervical spinal cord with very subtle enhancement	Brain	6497	575	Near	6	None	None
2	1	50–54	Multiple enhancing lesions of left cerebellum, right dentate, left vermis, left medial occipital lobe	Brain	6571	755	Periphery/low dose	4	Glatiramer acetate	Glatiramer acetate
	2	50–54	Enhancing lesion of right posterior temporal lobe	Brain	6571	755	Periphery/low dose	31	Glatiramer acetate	Glatiramer acetate
	3	50–54	Minimally enhancing left frontal lesion	Brain	6571	755	Near	50	Glatiramer acetate	Glatiramer acetate
3	1	15–20	Non-enhancing T2 hyperintensity of right frontal lobe	Brain and spinal cord	5664 brain; 2983 cord	1577 brain; 225 cord	Near	19	None	None
3	1	15–20	Non-enhancing T2 hyperintensity of right temporal lobe	Brain and spinal cord	5664 brain; 2983 cord	1577 brain; 225 cord	Periphery/low dose	19	None	None
4		50–54	Non-enhancing T2 hyperintensity of thoracic spine	Brain	5333	273	Distant	28	Diroximel fumarate	Diroximel fumarate
5		45–50	Enhancing lesion of right frontal lobe	Brain	5745	790	Center/high dose	4	Dimethyl fumarate	Dimethyl fumarate
6		50–54	Enhancing lesion abutting the posterior lateral ventricles	Brain and spinal cord	5000 brain; 1301 cord	25 brain; 45 cord	Distant	36	Interferon beta	Interferon beta
7		60–64	Punctate enhancing lesion in the left parietal lobe	Brain and spinal cord	5000 brain; 3334 cord	25 brain; 2182 cord	Distant	25	None	None

Abbreviations: cGy = centigray. For location of lesion proximity to radiation field: Distant = radiation is far from the lesion, ie radiation of the spinal cord with brain lesion; near = radiation is close to the lesion, ie radiation of the right occipital/parietal lobes with lesion in the left frontal lobe (Fig. 3B); peripheral/low dose = lesion at the edge of radiation field with radiation delivered < 2000 cGy; center/high dose = lesion at the center of radiation field with highest radiation dose. *Age generalized to a range of 5 years to anonymize data.

### Characteristics of patients with new lesions

In the cohort of patients with new demyelinating lesions after radiation, there were a higher representation of ethnic minorities (3/7, 2 Hispanic, 1 Native American, 43%) versus those without new lesions (0/26, 0%, p = 0.0064). Otherwise, there were no statistically significant differences between those who did and did not develop new demyelinating lesions in the various clinical aspects we analyzed, including radiation dose and DMTs at the time of therapy. None of the patients with new lesions were on a high efficacy DMT (CD20 depletion agents, cladribine, natalizumab, and alemtuzumab), and only 3/7 patients (43%), were treated with any DMT. Further details are found in Table 3.

**Table 3:** Comparison of characteristics of patients with and without new demyelinating lesions.

		No new lesions (n = 26)	New lesions (n = 7)	p value
General	Race (n, %white)	26, 100%	4, 57%	0.0064
Radiation	Patients receiving spinal cord radiation (n, %)	17, 65%	3, 42%	0.3926
	Radiation dose cord (cGy, median, range)	2506 (30–4423)	2983 (1301–3334)	0.5985
	Patients receiving brain radiation (n, %)	16, 62%	5, 71%	1
	Radiation dose brain (cGy, median, range)	4999 (1661–7366)	5745, (5333–6571)	0.3136
Treatment	% on DMT (n, %)	8, 31%	3, 42%	0.661
	% on high efficacy DMT (n, %)	4, 15%	0	0.5552

Abbreviations: cGy, centigray, DMT, disease.

## Discussion

We found that MS patients who were treated with modern ionizing radiation to the CNS, had a good clinical outcome from an MS perspective post-radiation. No patients suffered a clear MS relapse or MS progression attributable to radiation. Despite the lack of clinical attacks or significant progression, 21% of the cohort had new demyelinating lesions post-radiation, although most were outside of the radiation volumes. A higher proportion of patients with new lesions post-radiation were of a minority ethnicity (43% vs 0%); otherwise, there were no statistically significant differences between the two groups.

These findings contrast with our previously published work from 2006, which demonstrated that external beam radiation may be associated with increased significant neurotoxicity in MS patients compared to those without demyelinating diseases [4]. In the historical cohort of 15 patients with MS treated with radiation, 6 patients developed neurotoxicity attributed to their radiation, leading to significant neurologic deficits such as progressive gait disturbance, progressive quadriparesis, and severe cognitive dysfunction, among others. Of these 6 patients with progressive neurotoxicity following radiation, 3 were treated before 1986, and only one patient was treated after 1995. A likely explanation for the difference between previous observations for MS patients treated with radiation and the current cohort is the enhanced conformity of modern radiation, resulting in focused high-dose regions confined to the target and improved sparing of surrounding normal tissues. Technological advances such as proton radiation similarly reduce excess dose; protons stop and deposit all their dose within the target volume, eliminating the exit dose traveling through healthy tissue. Following the previous manuscript demonstrating radiation-related toxicity in MS patients, many radiation oncologists subsequently either omitted radiation or used conformal techniques such as proton therapy when necessary.

Notably, none of our patients with new demyelinating lesions were on high-efficacy medication during their radiation treatments or at the time of new lesion development; only 3 patients were on DMTs at all. This is likely because our cohort was relatively older at the time of their radiation, with median age of 60 years. Our cohort of patients without new lesions also had a low rate of concurrent treatment with DMTs or high efficacy DMTs. The lack of definite clinical attacks in our cohort does not preclude the possibility of a clinical attack related to new inflammatory disease. Surveillance bias represents an important consideration when interpreting the observed frequency of new asymptomatic demyelinating lesions in this cohort. Patients undergoing radiation therapy for oncologic indications typically undergo more frequent MRI surveillance than patients with stable multiple sclerosis, driven by tumor type, treatment response, and institutional practice patterns. This increased imaging intensity may have increased detection of radiographic MS activity that would otherwise have remained clinically silent.

Quantitative assessment of imaging frequency (e.g., MRIs per patient‑year, median interval between scans, or timing of baseline to first post‑radiation MRI) was not feasible in this retrospective cohort, as imaging schedules were highly heterogeneous and largely determined by oncologic rather than neurologic factors. Accordingly, we avoid direct comparisons with population‑level MS disease activity rates and interpret these findings descriptively. The detection of new lesions in this study should therefore be understood in the context of enhanced surveillance rather than as definitive evidence of radiation‑associated excess inflammatory activity. Given the small number of radiographic events, heterogeneous imaging schedules, and non‑random censoring driven by oncologic surveillance, we did not pursue incidence‑based or time‑to‑event analyses, as these approaches would be underpowered and potentially misleading; findings are therefore presented descriptively.

Ionizing radiation also exerts many different effects on the immune system. The first is direct and indirect tumoricidal effect, causing cell death and antigen release, leading to increased inflammation to facilitate the clearing of cellular debris [7]. Radiation has long been considered proinflammatory, with increased systemic release of TNF-α and Il-1β [8]; this is integrated into the radiobiologic understanding of facilitating tumour destruction and local control of disease [9]. This may account for the appearance of new lesions consistent with new disease activity distant from the radiation treatment field. However, recent evidence particularly in the immunotherapeutic cancer care era suggests that low-dose radiation (<1 Gy, LDR) has an anti-inflammatory effect resulting from reduced inducible nitric oxide synthase (iNOS) expression and expression of anti-inflammatory cytokines such as IL-10 [10]. LDR has been used to treat inflammatory conditions such as osteoarthritis [11]. There is also limited early evidence in animal models that LDR may be helpful in neurodegenerative conditions that has been linked to neuroinflammation, such as Alzheimer’s and Parkinson’s diseases [12], [13], [14]. More studies are needed in this area to better characterize the effect of radiation on the immune and nervous systems.

Our study also found that all three of our patients of minority ethnicities had new demyelinating lesions after radiation. This is limited by our small sample size, so it is difficult to draw a strong conclusion. Ethnic differences in radiation related toxicity studies have not previously led to definitive conclusions, with significant barriers in the reporting of ethnicities and confounding factors such as socioeconomic effects15 [15]. Ethnic differences in MS is also studied, with Black Americans having a worse prognosis, with higher rates of both primary and secondary progressive disease compared to White Americans [16], [17]. As with studies of ethnic differences in radiation, however, it is difficult to account for socioeconomic, medical co-morbidities, and other potential confounders. This note is meant for hypothesis-generating only, as this small sample size is insufficient to draw conclusions.

This study’s limitations include the retrospective design and a potential referral bias at a tertiary referral site. We included only patients who underwent neurological evaluation and MRI with images available for review. This may have influenced the total number of cases identified. Despite vigorously applying the McDonald Criteria in establishing new demyelinating lesions, we cannot definitively rule out alternate aetiologies for these lesions, such as post-radiation vasculitis. However, it is notable that the lesion found at the centre of the radiation field, depicted in Fig. 3.D2 is quite convincingly consistent with a demyelinating lesion with a positive central vein sign. Despite being the largest study of this type to date, the sample size is still relatively small, which may limit the generalizability of the study. Another limitation of the study is that the radiation modality for all except one patient was protons. While results should be the same for other highly conformal techniques and similar radiation dosing, this study does not permit certainty in this regard. We defined a clinical relapse as typical demyelinating neurologic deficits with an associated MRI lesion in the correct location. There is a potential to miss events not captured in chart review or if imaging was not obtained. Lastly, by design, a significant proportion of our cohort, 15 patients (45%), had a malignant space occupying lesion in the CNS and subsequently had surgical resection of their tumour. Both the malignancy and cancer-directed treatments affected the neurologic status of the patient including various aspects of the EDSS. As a result, it was very difficult, if not impossible, to differentiate effects of the radiation, malignancy, treatment of malignancy, and MS disease on the resulting focal neurologic deficits noted.

In conclusion, we studied a cohort of MS patients undergoing ionizing radiation involving the CNS for any reason. We found no clinical worsening due to radiation but did find an excess of new asymptomatic MS inflammatory disease activity. This contrasts with our prior publication and at least in part can be attributed to the advances in radiation technology. In cases where radiation is indicated for a tumour in a patient with MS, it appears that radiation can be safely administered, although caution should be applied and dose to normal tissue minimized where possible. High efficacy DMT during and post radiation may offer an additional layer of protection against the development of new demyelinating lesions.

## Declaration of competing interest

The authors declare the following financial interests/personal relationships which may be considered as potential competing interests: Dr. Lachance reports grant funding from the National Institute of Health. Dr. Tobin reports grant funding from the National Institutes of Health, Merck, and the Mayo Clinic Center for Multiple Sclerosis and Autoimmune Neurology; he receives book royalties from Oxford University Press. The other authors report no disclosures. This is an open access article distributed under the terms of the CC-BY license (https://creativecommons.org/licenses/by/4.0/).
